# Effect of osteosynthesis, primary hemiarthroplasty, and non-surgical management for displaced four-part fractures of the proximal humerus in elderly: a multi-centre, randomised clinical trial

**DOI:** 10.1186/1745-6215-10-51

**Published:** 2009-07-08

**Authors:** Stig Brorson, Bo Sanderhoff Olsen, Lars Henrik Frich, Steen Lund Jensen, Hans Viggo Johannsen, Anne Kathrine Sørensen, Asbjørn Hrobjartsson

**Affiliations:** 1 Dept of Orthopaedic Surgery, Herlev University Hospital, Herlev, Denmark; 2 Dept of Orthopaedic Surgery, Aarhus University Hospital, Aarhus, Denmark; 3 Dept of Orthopaedic Surgery, Odense University Hospital, Odense, Denmark; 4 Dept of Orthopaedic Surgery, Farsø Clinic, Farsø, Denmark; 5 The Nordic Cochrane Centre, Rigshospitalet, Copenhagen, Denmark

## Abstract

**Background:**

Fractures of the proximal humerus are common injuries and account for 4–5 percent of all fractures, second only to hip and wrist fractures. The incidence is positively correlated with age and osteoporosis, and is likely to increase. Displaced four-part fractures are among the most severe injuries, accounting for 2–10 percent of proximal humeral fractures. The optimal intervention is disputed. Two previous randomised trials were very small and involved a noticeable risk of bias, and systematic reviews consequently conclude that there is inadequate basis for evidence-based treatment decisions. We aim to compare the effect of osteosynthesis with angle-stable plate with non-surgical management, and the effect of primary hemiarthroplasty with both osteosynthesis and non-surgical management.

**Methods/Design:**

We will conduct a randomised, multi-centre, clinical trial including patients from ten national shoulder units within a two-year period. We plan to include 162 patients. A central randomisation unit will allocate patients. All patients will receive a standardised three-month rehabilitation program of supervised physiotherapy regardless of treatment allocation. Patients will be followed at least one year. The primary outcomes will be the overall score on the Constant Disability Scale, and its pain subscale, measured at 12 months. A blinded physiotherapist will carry out the assessments. Other secondary outcomes are Oxford Shoulder Score, and general health status (Short Form-36).

## Background

Fractures of the proximal humerus are common injuries and account for 4–5 percent of all fractures[1,2]. The incidence is expected to increase due to its association with advanced age and osteoporosis [3-5]. Displaced four-part fractures are among the most severe injuries accounting for 2–10 per cent of all proximal humeral fractures [6-8] and for about one out of four operated proximal humeral fractures[9].

**Figure 1:**
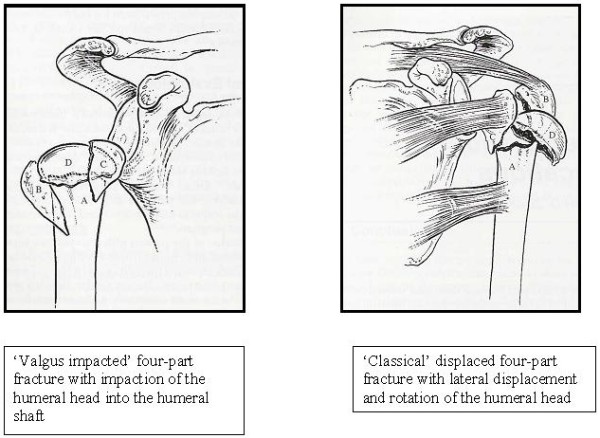
**Two patterns of displaced four-part fractures**. Reprinted from Murray and Zuckerman [18].

The optimal treatment for displaced four-part fractures is disputed. Several surgical techniques have been suggested including transcutaneus reduction, external fixation, hemiarthroplasty or open reduction and internal fixation with Kirschner wiring, tension-band wiring, transosseous suturing, plate fixation, intramedullary rod fixation, screw fixation, or, most recently, angle-stable plates. Numerous clinical series have been published, but most techniques have shown unsatisfactory results except in 'valgus impacted' fracture patterns (Fig. 1) or in selected younger populations [10-14].

Two previous very small randomised clinical trials compared treatments for displaced four-part fractures. Stableforth[8] compared arthroplasty with non-surgical management on 32 patients. The result tended to favour surgery. Hoellen[15] compared arthroplasty with osteosynthesis on 30 patients, but found no statistically significant difference in outcome. Both trials had low power, and did not report clearly adequate concealment of patient allocation or blinding procedures. The trials were analysed and summarised in a Cochrane review[16] that emphasised that the limited evidence available does not even confirm that surgery is preferable to conservative treatment in displaced fractures. Other systematic reviews have concluded that published data are inadequate for evidence-based decision making[6,17].

### Aims

Our primary aim is to compare the effect of osteosynthesis with angle-stable plate with non-surgical management for displaced four-part fractures in elderly. We also aim to compare the effect of primary hemiarthroplasty with both osteosynthesis and with non-surgical management.

Our secondary aim is to study prognostic differences between two pathoanatomical patterns of displaced four-part fractures: 'valgus impacted' four-part fractures and 'classical' four-part fractures (Fig. 1) [18].

## Methods/design

A randomised, multi-centre, national, clinical trial.

### Inclusion criteria

We will include patients diagnosed with a displaced four-part fracture within a two-year recruitment period from ten Danish hospitals providing advanced shoulder service. Eligibility criteria will be: age ≥ 60 years, patients mentally alert and physically fit (ASA-group 1–3) for surgery and rehabilitation.

The diagnoses of four-part fractures will be based on plain radiographs (at least two perpendicular views) and any available supplemental imaging material (CT or 3D CT). At least two consenting experienced shoulder surgeons should classify the fracture as a displaced four-part fracture. The timing of the classification, and the subsequent recruitment procedure, will be such that an operation can be conducted within two weeks of injury.

### Exclusion criteria

We will exclude patients with fracture-dislocations, head-splitting fractures, previous shoulder surgery, chronic shoulder pain, abuse problems, and patients unable to understand instructions in Danish and follow the rehabilitation protocol at their local hospital.

### Randomisation and concealment of allocation

After obtaining informed consent patients will be included in the trial and randomly allocated to three groups of equal size:

• Non-surgical management (physiotherapy and self-training).

• Osteosynthesis with angle-stable plate followed by physiotherapy and self-training. If osteosynthesis during the operation is regarded surgically impossible by the operating surgeon the operation will be converted to primary modular hemiarthroplasty.

• Primary modular hemiarthroplasty followed by physiotherapy and self-training.

The randomisation sequence will be computer generated. A remotely placed randomisation unit, otherwise not involved in the study, will irreversibly include patients into the trial before allocating them to treatment groups, communicating the result to the recruiting surgeon through telephone.

After randomisation the patients and surgeon involved will be informed about the patient's allocation to either non-surgical management or to surgical management, but they will not be informed about the type of surgery. The surgical procedure (osteosynthesis or hemiarthroplasty) will be revealed to the operating team just before the operation.

### Interventions

Surgery will be performed within 14 days of injury by a trained shoulder surgeon familiar with both surgical interventions. Operative techniques follow standardised instructions.

All patients will receive a standardised rehabilitation program of supervised physiotherapy and instructed self-training regardless of treatment allocation. The local physiotherapists will be involved in the assessment of the outcome after 12 months and will record compliance with the rehabilitation program.

### Evaluation

All patients will be followed actively for one year post injury. They will be seen by the responsible shoulder-surgeon in the out-patient clinic at two weeks, six months and 12 months. All patients will be radiographed (at least two plain perpendicular radiographs) prior to each visit. Clinical outcome assessment will be performed after six and 12 months by a blinded central physiotherapist not involved in the rehabilitation of the patient. In addition, the local physiotherapists will assess outcome after 12 months. The evaluation procedure will follow a standardised protocol.

Patients will be followed up after three years. No clinical assessment will be carried out, but data on complications that may have developed after the 12 months evaluation is extracted from the clinical databases at each hospital (discharge letters, discharge diagnosis, and if needed journal notes).

### Outcome measures

Clinical outcome is evaluated using an observer administered shoulder-specific score (Constant Disability Scale)[19,20], a patient administered shoulder-specific questionnaire (Oxford Shoulder Score)[21], and a patient administered general health status questionnaire(Short Form-36)[22,23].

The primary outcomes are

• Constant Disability Scale – total score (0–100 points) at 12 months

• Constant Disability Scale – pain subscale score (0–15 points) at 12 months

Secondary outcomes are

• Shoulder function. Oxford Shoulder Score – total score (12–60 points)

• General health. Short Form-36 questionnaire – total score (0–100 points)

### Patient drop-out and protocol violations

Patients who drop-out of the trial will be recorded, and the reason for drop-out will be noted. Patients that do not comply with self-training and physiotherapy will be recorded. Patients that cross-over from one treatment group to another (non-surgical management to secondary hemiarthroplasty or osteosynthesis to primary hemiarthroplasty) will be recorded.

### Side-effects reporting

We will record any case of deep infection, nerve injury, implant malposition, heterotopic ossification, avascular necrosis, malunion, nonunion, instability, dislocation, implant removal and revision surgery. We also record any case of pulmonary embolism, other medical complications, and death by any cause during the follow-up period.

### Statistical analysis plan

#### Sample size and power calculation (ANOVA)

To have an 80% chance of detecting as significant (at the 5% level) a ten point difference or more in mean total Constant score between the three groups, with an assumed standard deviation of 15 points and a loss to follow up of 10 patients in each group, a total of 162 patients is required.

#### Data-analysis

No interim data-analysis will be carried out. The data-analysis will be conducted by a biostatician at the end of the follow-up period.

Patients randomised to the non-surgical management group but who cross-over and undergo secondary surgery will primarily be analysed as they were randomised. Similarly, patients randomised to osteosynthesis but due to surgical technical difficulties are treated with primary hemiarthroplasty, will be analysed as randomised.

Patients that drop-out after the six months evaluation will be included in the analysis based on six months data ('last observation carried forward'). Drop-out of patients before the six months evaluation cannot be included in the analysis.

Descriptive statistics (mean and standard deviations, as well as median and 10 and 90 percentiles for continuous variables, and frequency and 95 percent confidence interval for binary variables) will be calculated for each of the three groups, and their relevant subgroups, at both time points. Relevant subgroups include:

• Valgus-impacted versus classical four-part fractures

• Cross-overs from non-surgical treatment to secondary hemiarthroplasty versus non cross-overs in non-surgical treatment group

• Cross-overs from osteosynthesis to primary hemiarthroplasty versus non cross-overs in osteosynthesis group.

Continuous and binary baseline characteristics will be tabulated according to the three treatment groups. No statistical testing will be performed, but relevant imbalances will be noted and reported. Baseline characteristics include: age, ASA Score, patient's treatment preference, surgeon's treatment preference and fracture pattern (valgus-impacted or classical four-part).

The primary analyses of effect will be two analyses of variance (ANOVA) based on the overall Constant scores, and the Constant pain subscale score, measured at 12 months. We will use the last observation carried forward approach, excluding patients only because of missing data, and analyse all patients according to their randomisation (intention-to-treat analysis).

If one or both of the ANOVA analyses are statistically significant, we will subsequently perform a pair-wise testing based on t-tests:

• Osteosynthesis versus non-surgical management.

• Hemiarthroplasty versus non-surgical management.

• Osteosynthesis versus hemiarthroplasty.

Thus, our protection against the risk of type 1 errors consists on a clear definition of the two primary outcomes and a single analysis ANOVA approach.

We will also conduct the following secondary analyses:

• The above analysis will be conducted for the following two subgroups: valgus-impacted and classical fractures respectively.

• In the osteosynthesis group we will conduct a subgroup analysis comparing patients with intraoperative change to hemiarthroplasty with those without.

• We will repeat the primary analysis as a mixed model for repeated measurements (including data from six months and the predefined covariates: age, ASA-Score, and treatment preference at baseline).

• The number of patients with Constant score ≥ 70 at month 12 for each group (and subgroup) will be compared with Chi-square tests.

The same analyses (except the third) will be performed for the other outcomes (Oxford Shoulder Score, Short Form-36).

Finally, we will perform a per protocol effect analysis in which only patients that were compliant with physiotherapy and training (attending > 75% physiotherapy sessions judged by physiotherapist) will be included.

The same analyses will be performed for the other outcomes (Oxford Shoulder Score, Short Form-36).

A three year follow-up analysis will be reported in a separate publication. The analyses will compare number of patients in each group that died, received shoulder surgery, etc.

#### Blinding

The trial will involve several levels of blinding:

• Patients, and all persons involved with the trial, except the operation team, will be blinded to which operation was performed.

• A central physiotherapist blinded to patients' treatment will perform the clinical evaluation at 6 and twelve months. During evaluation patients will be wearing a shirt covering the shoulder and will have been asked not to disclose their treatment status to the physiotherapist, and the physiotherapists will be asked to avoid from actively exploring patients' treatment status. The blinding will be tested by asking the central physiotherapist to guess the patient's treatment allocation.

• By the end of the study, but prior to the data analysis, sub-classification in 'classical' and 'valgus-impacted' fracture patterns will be made by four experienced shoulder surgeons blinded to the patients' identity based on the baseline radiographs. Disagreement will be resolved by discussion.

• All three persons involved in the statistical analysis (statistician, SB, AH) will be blinded with respect to the treatment allocation, patient identity and surgeon identity when conducting the primary analysis. When conducting the additional statistical analysis the three persons will be blinded only to the identity of patients and surgeons.

#### Ethical issues

Each patient who wants to be part of the trial will be informed orally and in writing. Those that decide to join the trial will be asked to sign an informed consent form. The study protocol, including the informed consent form and patient information sheets have been approved by The Committee on Medical Research Ethics, The Capital Region of Denmark: Jr. H-C-2008-065. The processing of personal data has been be approved by the Danish Data Protection Agency.

### Research plan

Start date: April 2009

Finish date of recruitment: March 2011

Finish date of follow-up: March 2012

Submission date of paper: August 2012

## Competing interests

The authors declare that they have no competing interests.

## Authors' contributions

SB had the idea and drafted the protocol. All authors participated in the revision of the protocol. SB wrote the manuscript. All authors approved the final manuscript.
